# Changes in femoral anteversion after intramedullary nailing for pediatric femoral shaft fracture: a multicenter study

**DOI:** 10.1186/s12891-024-07566-z

**Published:** 2024-07-12

**Authors:** Jae Jung Min, Soon-Sun Kwon, Kibeom Youn, Daehyun Kim, Ki Hyuk Sung, Moon Seok Park

**Affiliations:** 1 Department of Orthopaedic Surgery, Seoul National University Bundang Hospital, 82 Gumi-Ro 173 Beon-Gil, Bundang-Gu, Sungnam, 13620 Gyeonggi Korea; 2 Department of Orthopaedic Surgery, Seoul National University College of Medicine, 103 Daehak-Ro, Jongno-Gu, Seoul, 03080 Korea; 3 Departments of Mathematics and Department of Artificial Intelligence, Ajou University, Gyeonggi, Korea; 4 Didim, Inc, Gyeonggi, Korea

**Keywords:** Pediatric femoral shaft fracture, Postoperative femoral anteversion, 3D reconstruction, Uncalibrated 2D radiographs

## Abstract

**Background:**

The rotational change after using a flexible intramedullary (IM) nail for femoral shaft fractures has been a concern for many surgeons. Recently, a statistical shape model (SSM) was developed for the three-dimensional reconstruction of the femur from two-dimensional plain radiographs. In this study, we measured postoperative femoral anteversion (FAV) in patients diagnosed with femoral shaft fractures who were treated with flexible IM nails and investigated age-related changes in FAV using the SSM.

**Methods:**

This study used radiographic data collected from six regional tertiary centers specializing in pediatric trauma in South Korea. Patients diagnosed with femoral shaft fractures between September 2002 and June 2020 and patients aged < 18 years with at least two anteroposterior (AP) and lateral (LAT) femur plain radiographs obtained at least three months apart were included. A linear mixed model (LMM) was used for statistical analysis.

**Results:**

Overall, 72 patients were included in the study. The average patient age was 7.6 years and the average follow-up duration was 6.8 years. The average FAV of immediate postoperative images was 27.5 ± 11.5°. Out of 72 patients, 52 patients (72.2%) showed immediate postoperative FAV greater than 20°. The average FAV in patients with initial FAV > 20° was 32.74°, and the LMM showed that FAV decreased by 2.5° (*p* = 0.0001) with each 1-year increase from the time of initial trauma.

**Conclusions:**

This study explored changes in FAV after femoral shaft fracture using a newly developed technology that allows 3D reconstruction from uncalibrated 2D images. There was a pattern of change on the rotation of the femur after initial fixation, with a 2.5° decrease of FAV per year.

## Background

Femoral shaft fracture is a common injury that accounts for approximately 1.6% of all bone injuries in children [1]. The treatment of femoral shaft fractures varies according to patient age, ranging from conservative treatments, such as Pavlik harness and hip spica cast, to internal fixation using an intramedullary nail or plate. Flexible intramedullary (IM) nails can be adopted for the treatment of femoral shaft fractures from 24 months to maturity and are commonly recommended in children between ages 6 and 11 years [1].

The acceptable range of angulation also varies with age, ranging from 5 to 30º varus or valgus, 10 to 30º anterior or posterior angulation, and 10 to 15 mm shortening [1]. While angulation and shortening are easily assessed intraoperatively, the rotational change after placement of a flexible IM nail for a femoral shaft fracture has been a question for many surgeons; only a few methods, such as comparing the appearance of the lesser trochanter to the non-injured side, are feasible for checking femoral anteversion during surgery.

The radiographic methods used to determine the rotational profile include postoperative torsional computed tomography (CT) and three-dimensional reconstruction using a biplanar system (EOS®). However, performing these studies solely to evaluate the postoperative rotational profile may seem excessive [2–5]. A study performed in 1994 confirmed poor remodeling potential in torsional deformity in pediatric femoral shaft fractures; however, only a few studies on rotational malalignment in femoral shaft fractures have been performed since then [6].

Recently, a novel technology that allows three-dimensional reconstruction of the femur from two-dimensional anteroposterior (AP) and lateral (LAT) plain radiographs has been developed [7]. This technology allows reliable and valid measurement of the rotational profile without extraneous examination, as plain postoperative AP and LAT radiographs are included in routine postoperative care.

Torsional malalignment can occur during any treatment of femoral shaft fractures, and the developed mobile application is applicable to all follow-up radiographs, regardless of the treatment option. However, this retrospective study focused on patients treated with flexible IM nails. In this study, we measured postoperative femoral anteversion (FAV) in patients diagnosed with femoral shaft fractures who were treated with flexible IM nails and investigated the changes in FAV with age.

## Methods

This study was approved by the Institutional Review Board of Seoul National University Bundang Hospital (IRB No. B-2007–622-111). Procedures were performed in accordance with the Helsinki Declaration of 1975, revised in 2000. The need for informed consent was waived owing to the retrospective nature of this study, which only used existing radiographic images. The waived consent was confirmed by the institutional review board of Seoul National University Bundang Hospital.

### Patient selection and study design

This study used radiographic data collected from six hospitals in South Korea. The participating hospitals were tertiary regional centers for pediatric trauma. From each hospital, patients who were admitted to each institute and treated for femoral shaft fractures were identified by searching for the keywords “femoral shaft fracture” and the International Classification of Diseases (ICD) code S7208.

**Fig. 1:**
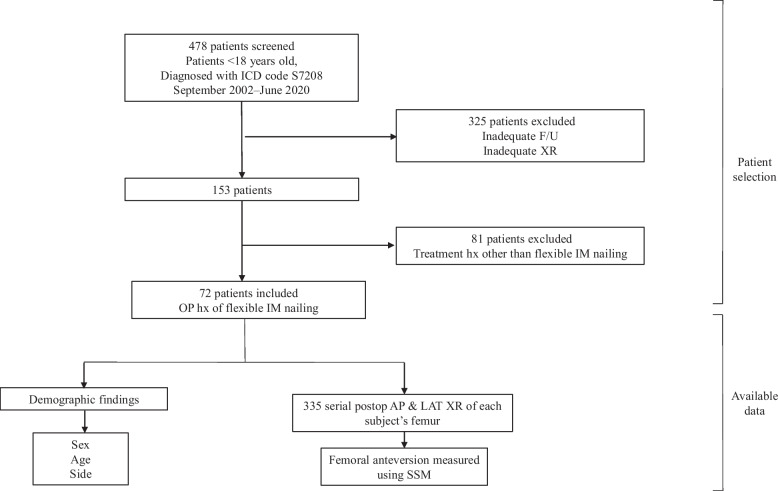
Flowchart for participant inclusion. F/U = Follow-up; hx = history; IM = intramedullary; AP = anteroposterior; LAT = lateral; SSM = statistical shape model

Patients diagnosed with femoral shaft fractures between September 2002 and June 2020 and pediatric patients aged < 18 years with at least two anteroposterior (AP) and lateral (LAT) femur plain radiographs obtained at least three months apart were included. Patients with radiographic images that lacked full coverage of either proximal or distal femur were excluded from analysis. Among the included patients, those with a history of treatment for fractures using flexible IM nailing were selected (Fig. 1). Each patient’s serial radiographs immediately after surgery up to their last follow-up were subjected to 3D (three-dimensional) reconstruction to measure the FAV.

### System overview

**Fig. 2:**
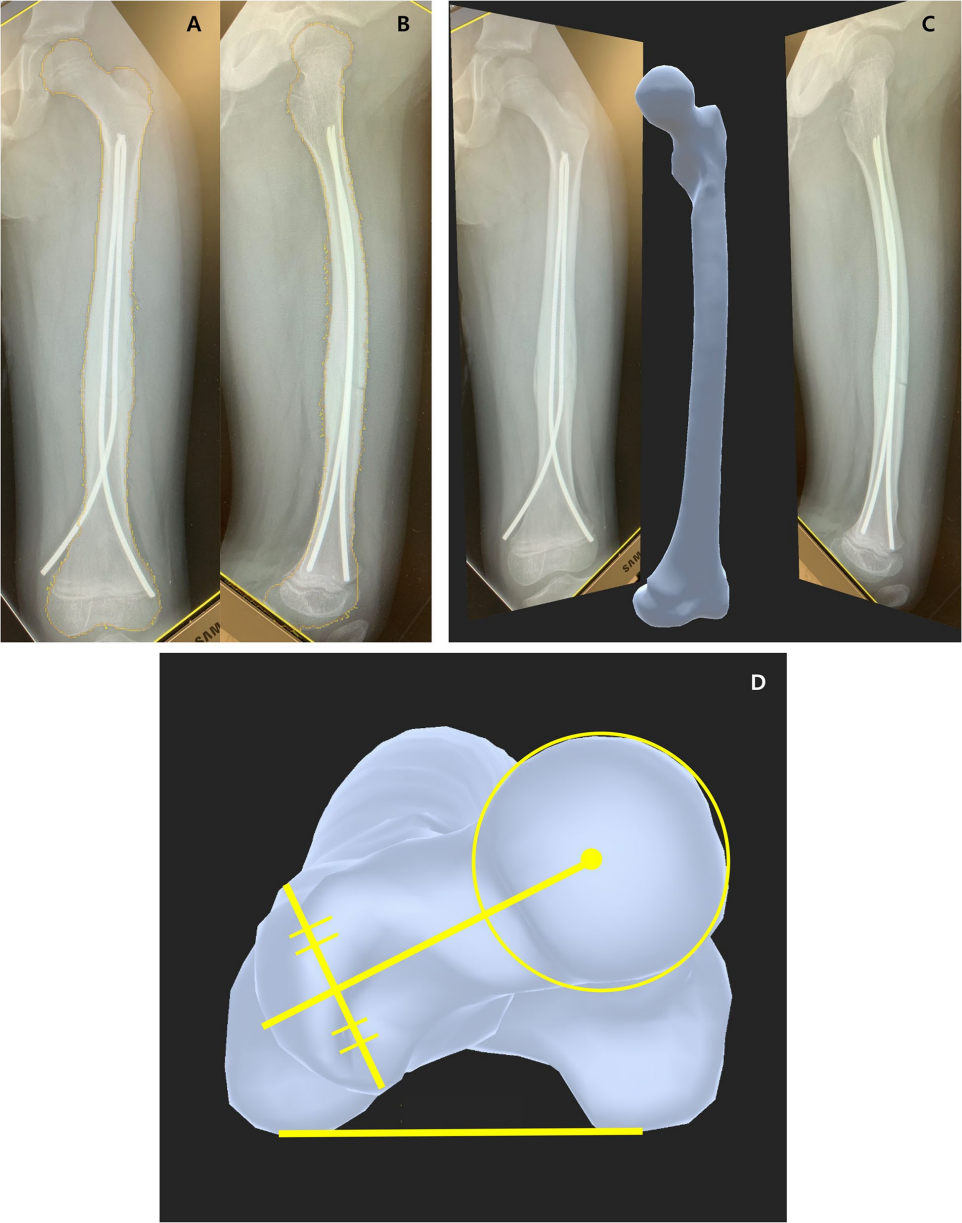
Three-dimensional reconstruction using the statistical shape model (Femora®). This is performed by outlining the bony cortices, allowing accurate measurement even when metal implants are present (**A**, **B**). A three-dimensional reconstruct is obtained from two-dimensional uncalibrated plain radiographs (**C**). Femoral anteversion can be measured by measuring the angle between a line connecting the femoral head center and neck, and another line connecting the posterior femoral condyles (**D**)

The developed 3D reconstruction system utilizes a statistical shape model (SSM) constructed from existing CT data. The basic principle is as follows: 1) Construct an SSM using existing CT data, 2) generate a 3D shape based on the SSM and find a shape that fits the patient's 2D images, and 3) measure the FAV using the generated 3D shape. The shape of the femur was deduced from the 2D images, and the reconstructed model was iteratively compared with the SSM until the best-fit model was constructed [7, 8]. The program was developed as a mobile application with a user-friendly interface (Femora®, Didim, Inc., South Korea) (Fig. 2A-C).

### Reliability testing & measurements

Before measurement, a consensus-building session was held among examiners with 3, 6, and 22 years of orthopedic experience. The proximal landmarks for measurement were defined as the lines connecting the center of the femoral head and the bisecting point of the greater trochanter. The distal landmark was defined as the line connecting the two posterior condyles of the distal femur (Fig. 2D).

Following the established consensus regarding the indices, we conducted a reliability test prior to the primary measurements. The sample size estimation showed that radiographs of 36 femurs (18 right and 18 left) were required for the assessment. Three authors performed the measurements independently. Interobserver reliability was determined using intraclass correlation coefficients (ICCs). For intraobserver reliability, one of the authors repeated the measurements 4 weeks after the primary measurements.

After the reliability testing, one of the authors measured the FAV on all serial postoperative radiographs of the selected patients for statistical analysis.

### Statistical analysis

The ICCs and their 95% confidence intervals (CIs) were used to summarize the interobserver reliabilities of the radiographic measurements and were calculated in the setting of a linear mixed model, assuming a single measurement and absolute agreement [9, 10]. With an ICC target value of 0.9, a Bonnett approximation was used with 0.2 set as the width of the 95% CI [11]. The minimum sample size required to detect clinically meaningful differences was 36.

Given the retrospective nature of the study, the selected patients varied in (1) follow-up duration, (2) age at initial presentation, (3) number of femoral AP and LAT images taken, and (4) the interval between follow-ups. To compensate for statistical errors that can occur with subject diversity, a LMM was adopted for statistical analysis [12]. The LMM was built to estimate the postoperative FAV of patients, with age at the time of operation and sex as fixed effects, and each subject and side as random effects. The covariance structure was assumed to be a variance component. Restricted maximum likelihood estimation was used to estimate the parameters of the LMM [12]. The models were accepted as valid for estimating responses using the Akaike Information Criterion (AIC) and Bayesian Information Criterion (BIC). Smaller AIC or BIC values were preferred for model selection. The model has a low AIC/BIC score. All the statistical analyses were performed using SAS version 9.4 (SAS Institute, Cary, NC, USA).

## Results

In total, 72 patients were enrolled after applying the inclusion and exclusion criteria. Of the enrolled patients, 48 were male and 24 were female, and 37 right and 35 left sides were assessed for FAV. The average patient age was 7.6 years, ranging from 2.2 to 16.8 years. The average follow-up was 6.8 years (range: 3–61 months) (Table 1).

**Table 1:** Summary of patient data (*n *= 72)

Parameters	Values
Patient information	
Sex (Male/Female)^a^	48/24
Side (Rt/Lt)^a^	37/35
Age at initial assessment^b^ (years)(range)	7.6 ± 3.0 (2.2 ~ 12.2)
Duration of follow-up^b^ (years)	6.8 ± 11.1

^a^Data are presented as the number of patients

^b^Data are presented as means and standard deviations

The interobserver reliability ranged from 0.95 to 0.98, with an ICC of 0.97. The intraobserver reliability ranged from 0.95 to 0.99, with an ICC of 0.98.

The average age in this population was 7.2 years old, ranging from 2.2 to 13.2 years. The number of follow-ups ranged from two to 16. Out of 72 included patients, 52 (72.2%) showed immediate postoperative FAV greater than 20°. The average FAV immediately after IM nail was 32.7 ± 8.3°, and the average FAV at the last follow-up was 32.0 ± 10.1°. In the LMM analysis of 52 patients and 249 XRs with an FAV greater than 20°, the FAV decreased by 2.5° (*p* = 0.0001) with each 1-year increase from the time of initial trauma (Table 2).

**Table 2:** Improvement in femoral anteversion after IM nailing for femoral shaft fractures (initial FAV > 20°)

	Estimate	95% CI	p value
Intercept	40.50	36.73 to 44.25	< 0.0001
Age (y)	-0.97	-1.42 to -0.52	< 0.0001
Duration (/y)	-2.54	-3.74 to -1.34	< 0.0001
Male sex	-1.14	-3.72 to 1.43	0.38
Right side	2.45	0.000032 to 4.90	0.05

*IM* Intramedullary, *CI* Confidence interval

## Discussion

This study included 72 patients treated with flexible IM nails for femoral shaft fractures. When the immediate FAV was greater than 20°, the FAV decreased by 2.5° each year after the initial surgery. Although the initial XR screening was conducted in all patients below 18 years old, to maximize the selection pool, the actual inclusion of the patients was limited to those around 12 years old, for flexible IM nail is not a conventional treatment option for those after this age.

The FAV is greatest at infancy, ranging approximately 40–45°, and gradually decrease and reach 15–20° by age 10 [13]. Whether this concept applies to post-traumatic femoral shaft fractures has rarely been discussed [6]. The acceptable rotational range is arbitrary. A deviation of the rotation profile within 10º from the contralateral side is considered acceptable, with a torsional difference of < 15º indicating torsional malalignment [14]. Another study asserted a functionally normal range of 10–15º, but a rotational deviation > 25° was also well-tolerated [6, 15]. Torsional malunion with FAV > 15° compared to contralateral side after flexible IM nailing is reported to range from 28 to 47%, depending on the institution [14, 16].

The results of a previous study confirmed the little postoperative changes on FAV in pediatric femoral shaft fractures, which differs from the findings of this study [6]. The result of this study shows another possibility on the femoral rotation after trauma with a changing pattern of 2.5° per year.

**Fig. 3:**
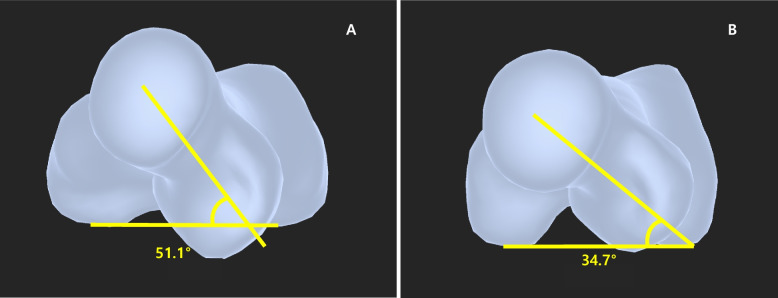
An example of a patient with femoral anteversion measured using the statistical shape model. **A** At the age of 5.6 years, a flexible IM nail was placed for the treatment of left femoral shaft fracture. FAV at the time of surgery was 51.1°. **B** At his last follow-up at the age of 9.7 years, the FAV improved to 34.7°. IM = intramedullary; FAV = femoral anteversion

Methods to assess FAV after the treatment of femoral shaft fractures in a clinical setting other than CT are lacking. Davids et al. evaluated torsional profiles in patients with femoral shaft fracture treated conservatively using CT, providing valuable results on changes in rotational deformities in pediatric femoral shaft fractures. [2, 3, 6] However, further assessment since Davids et al. was void, possibly because of the burden of taking CT scans. Therefore, a novel 3D reconstruction tool using uncalibrated images was used to assess the FAV in this study. The intra- and inter-rater reliabilities showed excellent ICCs. Assessing the FAV using an SSM falls within the scope of the routine follow-up process, as AP and LAT radiographs are required for follow-up of bone union. In addition, 3D reconstruction using CT in patients with IM nails is inaccurate because of the presence of metallic artifacts. 3D reconstruction using the SSM was performed by outlining the bony cortices which allows accurate measurements even when metal implants are present (Fig. 2A-D, 3).

This study has some limitations. First, it was a retrospective study. Therefore, a universal protocol for follow-up and radiographic imaging was lacking. The number of postoperative follow-ups among the participants was heterogeneous, ranging from one to 16. To overcome this limitation, an LMM was used. Second, due to the design of the study, the analysis of the unaffected side of femur was not feasible. In future studies, if sufficient data can be collected with radiographs of both femurs, a more precise analysis with patients selected for FAV difference greater than 15 ~ 20° may be done. Secondly, during the course of the follow-ups after surgery, most of the included patients lacked XRs on the opposite side. In order to fully assess the cause of changes to the FAV, comparison with the contralateral side is essential. However, owing to the inherent design of the study, comparing the fractured femur with the other side could not be done. In our institute, IM nails are removed at around 1 year after the surgery. The effect of removal on the changes to the FAV could not be assessed in this study. A prospective study may be needed for assessment of the effect of hardware removal on the FAV. In addition, whether the changing pattern of FAV is due to the patients’ remodeling potential could not be assessed in this study.

## Conclusions

This study explored changes in the FAV after femoral shaft fracture using a newly developed technology that allows 3D reconstruction using uncalibrated 2D images. There was a pattern of change on the rotation of the femur after initial fixation, with a 2.5° decrease of FAV per year.

## Data Availability

The datasets used and/or analysed during the current study are available from the corresponding author on reasonable request.

## Abbreviations

AIC: Akaike Information Criterion
AP: Anteroposterior
BIC: Bayesian Information Criterion
CT: Computed tomography
Cis: Confidence intervals
FAV: Femoral anteversion
ICD: International Classification of Diseases
ICCs: Intraclass correlation coefficients
IM: Intramedullary
LAT: Lateral
LMM: Linear mixed model
SSM: Statistical shape model
